# Comparative Study of the Cytotoxicity and Genotoxicity of Alpha- and Beta-Asarone

**DOI:** 10.3797/scipharm.1204-21

**Published:** 2012-05-31

**Authors:** Pascal Unger, Matthias F. Melzig

**Affiliations:** Institut für Pharmazie, Freie Universität Berlin, Königin-Luise-Straße 2+4, D-14195 Berlin, Germany.

**Keywords:** Asarone, HepG2-cells, Cytotoxicity, Genotoxicity, Micronucleus test

## Abstract

The cytotoxicity of alpha- and beta-asarone was investigated with the BrdU assay in HepG2-cells. Alpha-asarone was found to be more toxic than beta-asarone after 24 hours of treatment. Investigation of the genotoxicity using the micronucleus assay in the HepG2-cell system showed that only after metabolic activation by a liver microsomal preparation, beta-asarone was able to induce micronuclei at concentrations higher than 50 μg/mL.

## Introduction

Studies of the genotoxicity of phytoherbal preparations or isolated natural compounds are needed to in order to demonstrate their safety, and are prerequisites for approval procedures. For these studies, short-term *in vitro* tests to identify genotoxicants are necessary. Established cell lines can be used to determine cytotoxicity, but for the investigation of genotoxicity, theirapplication is rather limited.

Cell lines of liver origin are widely used in biomedical research involving xenobiotic metabolism including genotoxicity studies. The HepG2 cell line is one of the most often used cell systems in xenobiotic and metabolism research, which is biochemically and physiologically characterized in detail. This cell line is also qualified for the detection of environmental and dietary genotoxicants [1]. This cell line expresses a wide spectrum of phase I enzymes such as cytochrome P450 (CYP) 1A1, 1A2, 2B, 2C, 3A, and 2E1, arylhydrocarbon hydrolase, nitroreductase, *N*-demethylase, catalase, peroxidase, NAD(P)H:cytochrome *c* reductase, cytochrome P450 reductase, and NAD(P)H, Quinone oxidoreductase and phase II enzymes such as epoxide hydrolase, sulfotransferase, glutathione *S*-transferase (GST), uridine glucuronosyl transferase, and *N*-acetyl transferase. It has been demonstrated that cultivated HepG2 cells contain mixed-function oxidases [2]. Nevertheless, this cell line is not able to express all enzymes required for the activation of all test substances compared to the conditions *in vivo*. This problem can be solved using an exogenous activation mixture containing mammalian microsomal enzymes [3]. The most appropriate endpoint for experiments with HepG2-cells appears to be micronucleus induction [4].

Based on new regulatory requirements for pharmaceutical preparations made from plants of the genus Asarum, which also contain aristolochic acids [5], it would be interesting to know if there were also a toxicological risk induced by the accompanying components in the essential oils, like alpha-asarone. The genotoxicity of beta-asarone is confirmed [6], for alpha-asarone divergent results are published. In this study we investigated the cytotoxicity and genotoxicity of both alpha- and beta-asarone in HepG2 cells.

## Results and Discussion

**Fig. 1:**
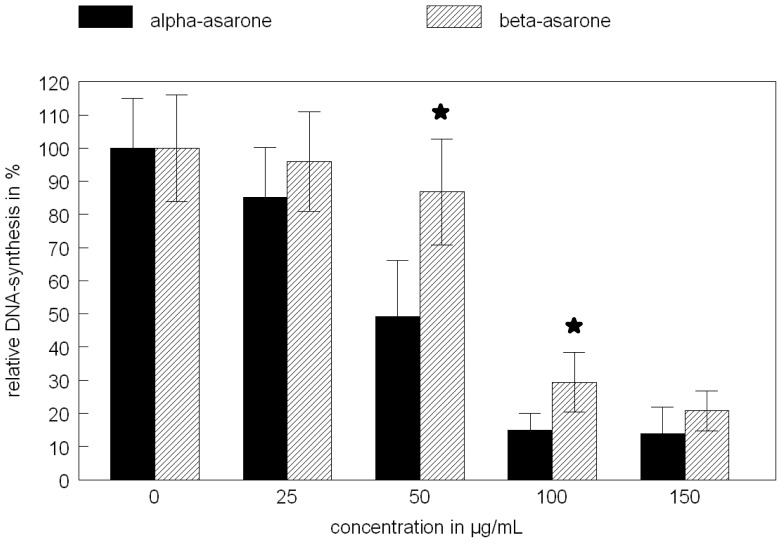
Cytotoxicity of alpha- and beta-asarone in HepG2 cells determined by the BrdU-assay. (★ significant difference compared to the control (no asarones) according to Mann-Whitney U test, p < 0.05, n= 3 with 6 parallel samples)

In HepG2-cells, alpha- and beta-asarone show a different cytotoxicity in the BrdU-assay without metabolic activation, as demonstrated in Fig. 1. The explanation for this result may be due to the different metabolisms of both isomers by the HepG-2 cells. The major metabolite of the asarones in hepatocytes was identified by liquid chromatography-mass spectrometry as 2,4,5-trimethoxycinnamic acid [7]. The results of the BrdU-assay to measure the cytotoxicity of both isomers cannot be correlated with the genotoxic assay, because 2,4,5-trimethoxycinnamic acid is not genotoxic. Data about its cytotoxicity are not yet published. Only beta-asarone is additionally metabolized via formation of epoxides, and by that it shows genotoxicity [7, 8]. This different metabolism of both enantiomers is demonstrated by the different results obtained in the micronucleus assay (Tab. 1). As well as with or without metabolic activation of both enantiomers, only beta-asarone shows an increase in the micronucleus formation in HepG2-cells, whereas alpha-asarone was inactive in the used cell system. The stronger metabolism of alpha-asarone in comparison to beta-asarone is reported [9]. That means the metabolite 2,4,5-trimethoxycinnamic acid accumulates in the cell culture and is responsible for the higher cytotoxicity in comparison to beta-asarone, which is further metabolized to genotoxic products with lower cytotoxicity than 2,4,5-trimethoxycinnamic acid. The data from Tab. 1 clearly show that the addition of a microsomal liver preparation (S9-mix) increases the toxification of beta-asarone in comparison to alpha-asarone, which resulted in an enhanced formation of micronuclei. The metabolic capacity of the HepG2 cells alone is insufficient to induce the micronucleus formation and the results from both isomers are comparable. The addition of the S9-mix also enhances the toxicity of both alpha- and beta-asarone, but in the same dimension (data not shown). These results clearly indicate that there is a different metabolism of these compounds based on their structural properties, resulting in different cytotoxicity and genotoxicity of the enantiomers.

## Conclusion

After compiling the results, both enantiomers of asarone show cytotoxicity, but only beta-asarone induces genotoxicity. On the other hand, alpha-asarone is more cytotoxic. For pharmaceutical preparations based on plants from the genus Asarum, which also contain essential oils with asarones, there is obviously no genotoxic risk based on the content of alpha-asarone, but the cytotoxicity of this compound should be taken into account. The transfer of these *in vitro* results on previously used drugs containing asarones requires the absence of beta-asarone. For this purpose, an analytical method is necessary which separates both isomers of asarone and detects low concentrations of the compounds. The values for the daily intake of alpha-asarone containing preparations should be regulated according to the TTC concept [10], i.e. the limit is fixed at 1.5 μg/day. Any drugs containing traces of beta-asarones should not be used for phytoherbal treatment.

## Experimental

### Materials

#### Cell lines & culture medium

HepG2 cells were obtained from DSMZ Braunschweig, Germany, the cell culture media and the fetal calf serum (FCS) were supplied from Biochrom AG, Berlin.

#### Reagents & kits

Alpha- and beta-asarone and all other chemicals were purchased from Sigma-Aldrich, Steinheim. The Cell Proliferation ELISA-kit (BrdU colorimetric) was obtained from Roche Diagnostics GmbH, Mannheim, the S9-mix (Sprague-Dawley rats, suspended in 0.154 KCl) from TRINOVA, Gießen. The plasticware for cell cultivation was supplied by Greiner bio-one, Frickenhausen.

#### Cell culture

The cells were cultivated in 75 cm^2^ flasks in RPMI 1640, supplemented with 10% FCS (5% CO_2_, 37°C) for 3 or 4 days before the experiments were started. Only passages from number 4 to 20 were used for the experiments. The cells were counted for the inoculation by Casy Cell Counter (Innovatis, Reutlingen). For detachment of the cells, EDTA-Trypsin-solution (0.02/0.05% in PBS) was used.

### Methods

For determination of the cytotoxicity, cell proliferation was measured using Cell Proliferation ELISA, BrdU (colorimetric) (Roche, Germany). HepG2 cells were seeded into 96-well plates at the concentration 20 000 cells/well and cultivated at 37°C, 5 % CO2 for 24 h. Subsequently, the cells were exposed to medium containing the asarones. After 24 h, 20 μL BrdU (100 μM) per well were added and incubated at 37°C for 2 h. The following procedures were carried out according to the manufacturer’s instructions. The changes in absorbance were recorded by a photometer (Tecan, Switzerland) at 450 nm wavelength. The DNA synthesis was calculated in comparison to the controls (with solvents) and shown in relation to the used concentration of alpha- or beta-asarone [11].

The determination of the micronucleus formation was performed according to a published protocol [12]. Briefly, in cell culture flasks (12.5 cm^2^) 37 500 cells were seeded in 2.5 mL RPMI medium + 10% FCS for 24 h. Appropriate dilutions of asarones (dissolved in DMSO) and DMSO (controls) were added to the cells directly. The cells were then cultivated for 24 h. When the S9-mix was used, the medium was changed and 625μL S9-mix per flask suspended in 1.875 mL medium was added for 6 h. After that, the medium was removed and fresh RPMI medium plus 3 μg/mL Cytochalasin B was added. After 24 h, the cells were detached from the flask bottom with EDTA-Trypsin-solution, diluted with medium, centrifuged (115 × g, 5 min) and the pellet was suspended in fresh medium. After counting the cells (Casy), a cell suspension with 2×10^5^ cells/mL fresh medium was prepared. 300 μL of the cell suspension were now centrifuged on a slide (Megafuge 1.0, Heraeus, with a special device for the centrifugation of cell suspensions on slides). For each culture flask two slides were prepared. After fixation in 90% methanol (−20°C) the slides were stained two times for 15 sec with a solution of acridine orange (0.0125% in PBS) and washed with PBS for 10 min. For determination of the number of micronuclei per 1 000 binuclear cells, the slides were counted by fluorescence microscopy (TMS-F, Nikon, 60x).

#### Statistical analysis

Data in tables and figures were presented as the mean ± SD. Differences between groups were assessed by the Mann-Whitney U test. A probability of p < 0.05 was considered significantly different. The test of normally distributed data was performed by the Shapiro-Wilk test. All tests were performed using the Statgraphics Plus 5.1 program.

## Figures and Tables

**Tab. 1:** Micronucleus formation in HepG2-cells induced by alpha- and beta-asarone in the absence and presence of rat liver S9-mix

	number of micronuclei per 1 000 binuclear cells				
**alpha-asarone**	0 μg/mL	50 μg/mL	100 μg/mL	150 μg/mL	200 μg/mL
without S9-mix	5.8 ± 1.3	8.0 ± 1.4	7.2 ± 3.0	5.3 ± 3.3	6.0 ± 0.8
with S9-mix	8.7 ±1.0	11.0 ± 2.0	12.3 ± 3.9	12.0 ± 2.5	10.7 ± 4.1

**beta-asarone**	0 μg/mL	50 μg/mL	100 μg/mL	150 μg/mL	200 μg/mL
without S9-mix	4.0 ± 3.2	3.8 ± 2.8	4.5 ± 1.9	4.3 ± 3.3	6.0 ± 1.4
with S9-mix	7.3 ± 1.9	10.8 ± 2.0*	19.0 ± 3.3*	19.0 ± 3.0*	17.7 ± 4.5*

* …significant difference compared to the control, U-test Wilcoxon-Mann-Whitney p< 0.05; mean ± SD, n= 2 or 3 with duplicate samples.

## References

[1] Knasmüller S, Mersch-Sundermann V, Kevekordes S, Darroudi F, Huber WW, Hoelzl C, Bichler J, Majer BJ (2004). Use of human-derived liver cell lines for the detection of environmental and dietary genotoxicants; current state of knowledge. Toxicology.

[2] Mersch-Sundermann V, Knasmüller S, Wu XJ, Darroudi F, Kassie F (2004). Use of a human-derived liver cell line for the detection of cytoprotective, antigenotoxic and cogenotoxic agents. Toxicology.

[3] Otto M, Hansen SH, Dalgaard L, Dubois J, Badolo L (2008). Development of an in vitro assay for the investigation of metabolism-induced drug hepatotoxicity. Cell Biol Toxicol.

[4] Schwab C, Kassie F, Qin HM, Sanyal R, Uhl IM, Hietsch G, Rabot S, Darroudi F, Knasmüller S (1999). Development of test systems for the detection of compounds that prevent the genotoxic effects of heterocyclic aromatic amines: preliminary results with constituents of cruciferous vegetables and other dietary constituents. J Environ Pathol Toxicol Oncol.

[5] Thiele A (2010). Bescheid des BfArM vom 20.07.2010 (75-3822-V12617-204289/10) zur Abwehr von Gefahren durch Arzneimittel, die unter Verwendung von Pflanzen der Familie der Aristolochiaceae mit der Gattung Asarum hergestellt werden.

[6] Kevekordes S, Spielberger J, Burghaus CM, Birkenkamp P, Zietz B, Paufler P, Diez M, Bolten C, Dunkelberg H (2001). Micronucleus formation in human lymphocytes and in the metabolically competent human hepatoma cell line Hep-G2: results with 15 naturally occurring substances. Anticancer Res.

[7] Hasheminejad G, Caldwell J (1994). Genotoxicity of the alkenylbenzenes alpha- and beta-asarone, myristicin and elimicin as determined by the UDS assay in cultured rat hepatocytes. Food Chem Toxicol.

[8] Oprean R, Tamas M, Roman L (1998). Comparison of GC–MS and TLC techniques for asarone isomers determination. J Pharm Biomed Anal.

[9] Björnstad K, Helander A, Hultén P, Beck O (2009). Bioanalytical investigation of asarone in connection with *Acorus calamus* oil intoxications. J Anal Toxicol.

[10] Munr IC, Renwick AG, Danielewska-Nikiel B (2008). The threshold of toxicological concern (TTC) in risk assessment. Toxicol Lett.

[11] Nitzsche D (2011). Untersuchungen zum genotoxischen Potenzial von Heilpflanzen am Beispiel der Aristolochiaceae. Dissertation.

[12] Fenech M (2007). Cytokinesis-block micronucleus cytome assay. Nat Protoc.

